# Thyroidectomy: A Standardized Surgical Technique with a Hybrid Energy Device to Minimize the Risk of Post-Operative Hemorrhage

**DOI:** 10.3390/jcm15155801

**Published:** 2026-07-24

**Authors:** Luca Risi, Paola Castiglione, Marta Noemi Monari, Walter Zuliani, Damiano Chiari

**Affiliations:** 1 Department of Biomedical Sciences, Humanitas University, Pieve Emanuele, 20072 Milan, Italy; luca.risi@humanitas.it (L.R.); paola.castiglione@humanitas.it (P.C.); 2 Laboratory of Clinical Analysis, IRCCS Humanitas Research Hospital, Rozzano, 20089 Milan, Italy; marta_noemi.monari@materdomini.it; 3 Department of General Surgery, Humanitas Mater Domini Clinical Institute, 21053 Castellanza, Italy; walter.zuliani@materdomini.it

**Keywords:** thyroidectomy, post-operative bleeding, peri-operative care, hybrid energy device, reoperation

## Abstract

**Background/Objectives**: Thyroidectomy is widely considered a safe procedure; however, several post-operative complications can occur, including post-surgery hemorrhage, which is considered a rare but life-threatening condition. Different strategies are employed before, during, and after surgery to minimize the risks. The aim of the present study is to describe a standardized patient management and surgical technique in thyroid surgery to prevent post-operative hemorrhage. **Methods**: Patients undergoing a thyroidectomy between January 2024 and June 2025 in a single center were included. Pre-operative strategies included blood pressure checks and thyroid function blood exams. Surgery was performed using a standardized technique and with a surgical energy device. Following the procedure, high blood pressure was corrected pharmacologically. **Results**: One hundred and twenty-three patients undergoing total thyroidectomy were included. Only one patient suffered from a post-operative hemorrhage, exhibiting an asymptomatic cervical hematoma that had been spontaneously drained one day after surgery. None of the patients required further surgeries for post-operative bleeding. **Conclusions**: With the application of standardized patient management and surgical techniques, zero cases of re-operation for post-operative bleeding were reported. This result strongly encourages the utilization of a standardized surgical technique to improve pre- and post-operative patient management and minimize the risk of life-threatening complications such as post-operative bleeding.

## 1. Introduction

Thyroidectomy is one of the most common surgical procedures in endocrinology and is used for both benign and malignant diseases.

Although it is widely considered a safe procedure, several post-operative complications can occur, with the most common including hypoparathyroidism, recurrent laryngeal nerve palsy (unilateral or bilateral), and bleeding [1]. Post-operative hemorrhages can arise immediately after a thyroidectomy, causing a hematoma that hampers the lymphatic and venous flow and resulting in airway obstruction as a consequence of laryngotracheal mucosal edema. Although post-operative bleeding after thyroidectomy is rare, with an incidence below 5% [2,3], it is a life-threatening condition, and almost all patients undergo re-operation when facing this complication [3,4]. Different factors can impact post-operative hematoma incidence rates, including male gender, hypertension, hyperthyroidism, bleeding disorders, operative time, and technical errors during surgery, such as the inadequate control of hemostasis [4,5].

Since the thyroid gland is highly vascularized, achieving optimal hemostasis is crucial in order to avoid serious complications. Over the years different strategies have been applied to decrease the rate of post-operative hemorrhage, such as the adequate control of arterial hypertension and hyperthyroidism before surgery [6], use of surgical energy devices and hemostats [7], and careful control of hemostasis during surgery via the Valsalva maneuver and drainage approaches [5,8,9].

By reporting the experience of the authors, this study presents a standardized surgical technique that includes various strategies for the prevention of post-operative hemorrhages following thyroidectomy.

## 2. Materials and Methods

### 2.1. Study Design

Between 2022 and 2023, our institution implemented a series of clinical practice modifications aimed at optimizing thyroid surgery outcomes. These refinements involved the pre-operative phase, the surgical technique, and post-operative management. This transition led to a comprehensive standardization of the clinical pathway, which was fully established by the end of 2023. Consequently, from January 2024 onwards, all patients followed an identical, standardized protocol.

After 18 months of clinical activity under this regimen, we performed a retrospective analysis of the prospectively collected data. All consecutive patients undergoing total thyroidectomy or hemithyroidectomy at Humanitas Mater Domini Hospital (Italy) from January 2024 to June 2025 were included in this study. Exclusion criteria were thyroglossal duct cyst excision and lateral lymph node dissection.

The surgical procedures were performed by the same two expert surgeons, acting as either the primary or assistant for all procedures. All clinical, surgical, and pathological data were prospectively recorded in a dedicated database.

The primary outcomes were the incidence of post-operative hemorrhage and the rate of reintervention for bleeding. Secondary outcomes included the readmission rate and the incidence of post-operative vocal cord palsy, hypoparathyroidism, seroma, and surgical site infection (SSI). Post-operative bleeding incidence was monitored from the day of surgery until the first follow-up visit, scheduled one week after discharge.

Patient characteristics are summarized using descriptive statistics: continuous variables are reported as means (standard deviation, SD), while categorical variables are presented as absolute numbers and percentages.

### 2.2. Surgical Technique

At our center, all patients undergo pre-operative exams, including blood pressure and thyroid function tests (TSH, fT3, and fT4 whenever the TSH value is below the limit). If uncontrolled arterial hypertension or hyperthyroidism is detected, an adequate pharmacological correction of these conditions is mandatory before surgery. Patients undergoing thyroidectomy for Graves’ disease should consume Lugol’s iodine solution for ten days before surgery.

Thyroidectomy is performed using a hybrid energy surgical device, combining ultrasonic and bipolar radiofrequency energy to simultaneously cut and seal tissues.

In fact, the device can be used for dissection and vessel closure via its ultrasonic “seal and cut” function, while the bipolar function “seal” can be used for hemostasis. In our experience, during dissection and vessel closure, the bipolar function (“seal”) is always used before the ultrasonic energy (“seal and cut”) to maximize hemostasis and blood vessel control, enabling operations in a clear surgical field. The approach is even more cautious when dealing with major thyroid vessels, such as the superior thyroid vascular pedicle, which is always tied off using ligation before sealing and cutting with the surgical device.

At the end of the surgery, hemostasis is carefully controlled by raising blood pressure and using a Valsalva maneuver performed by the anesthesiologist to check for any venous bleeding. Additionally, a hemostat is placed in the empty thyroid space (unilaterally for hemithyroidectomy and bilaterally for thyroidectomy), and a suction 9Fr drainage system is inserted.

During the post-operative course, blood pressure is checked manually by nurses, and if the systolic blood pressure tends to exceed 140 mmHg, it is lowered with intravenous clonidine or oral nifedipine.

The drain is usually removed one day after surgery, and the patient is discharged on the first or second post-operative day (POD).

The first follow-up appointment is scheduled one week after discharge.

## 3. Results

Throughout the study period, 123 patients undergoing total thyroidectomy or hemithyroidectomy were included. The patients’ characteristics are summarized in Table 1.

Most patients were female (77.2%), and the mean age was 57.3 years. A total of 64 patients (52%) underwent surgery for goiters, 27 (22%) for an indeterminate thyroid nodule (categories III and IV according to the Bethesda classification), 13 (10.6%) for a malignant nodule (categories IV and V according to the Bethesda classification), 4 (3.2%) for toxic adenoma, and 15 (12.2%) for Graves’ disease. The mean diameter of the major nodules was 34 (SD 17.6). A total of 67 patients (54.5%) were symptomatic, and 51 (41.5%) reported compression symptoms, while tracheal deviation was present on CT scans in 38 patients (30.9%). Retrosternal extension was reported in 15 patients (12.4%), and 35 patients (28.5%) were diagnosed with hyperthyroidism before surgery. The pre-operative TSH value was below the limit (0.4 mUI/L) in 20 patients (16.5%)—among them, only 2 presented with elevated fT4, while none exhibited increased fT3 or symptoms. The majority of patients underwent total thyroidectomy (83, 67.5%), and 40 (32.5%) underwent hemithyroidectomy. The mean operation time was 73 min.

All surgeries were performed by the same expert team of two surgeons according to the standardized technique using the hybrid energy surgical device.

As for the histology results (Supplementary Table S1), malignant disease was present in 18 patients (22.5%). Additionally, thyroiditis was reported in 23.1% of patient specimens.

The post-operative outcomes are detailed in Table 2.

Almost all patients were discharged on the first POD (115, 93.5%), while only seven (5.7%) were discharged on the second POD, and one experienced prolonged hospitalization until the seventh POD due to difficulties related to calcium blood level control. Only one patient suffered from post-operative hemorrhage, which presented as an asymptomatic cervical hematoma that had been spontaneously drained during the first POD without any additional intervention. No patient needed re-operation for post-operative bleeding. As for the other complications, post-operative paresthesia was reported in 13 patients (10.6%), and 11 (8.9%) exhibited hypocalcemia, but persistent hypoparathyroidism was reported in only one individual (0.8%). Monolateral post-operative cord palsy was reported after fibrolaryngoscopy in three patients (2.4%), persisting in only two of them. Minor complications such as seroma and SSI occurred in six (4.9%) and one patient (0.8%), respectively.

## 4. Discussion

In thyroid surgeries, postoperative hemorrhage is one of the most feared complications due to the risk of acute airway obstruction and the potential need for emergency surgical reintervention [2,3]. Although the incidence reported in the literature varies between 0.1% and 4.2% [10,11], this represents a potentially fatal event that necessitates rigorous management protocols. Our study results, reporting zero re-operations in 123 consecutive patients, suggest the importance of a standardized multimodal approach for the prevention of this complication.

Several factors contributed to this outcome. First, meticulous pre-operative patient optimization was performed, with a specific focus on the pharmacological control of hypertension and hyperthyroidism. Systemic hypertension is recognized as a primary risk factor for post-operative hematoma formation [5,12]. Aligning with recent evidence, we mandated the maintenance of systolic blood pressure values below 140 mmHg during and after surgery [11,12].

Technically, advanced energy devices represent a helpful adjunct for the treatment of intra-operative hemostasis. Hybrid energy surgical devices, integrating ultrasonic and bipolar technologies, enable effective vascular sealing [7,13,14]. Within our standardized approach, the sequential use of bipolar and ultrasonic functions is employed to refine hemostatic control, assisting in the reduction in operative time. However, technology does not replace classical surgical maneuvers. Consistent with the suggestions of Cernea et al. [6], we maintained the ligation of the superior thyroid pedicle and the systematic execution of the Valsalva maneuver before closure. This maneuver is fundamental for identifying occult venous bleeding that may only manifest with the increase in intrathoracic pressure during extubation [12,15]. Additionally, a hemostat was placed in the empty thyroid space.

Another point of discussion concerns the use of drains. Although their role in preventing hematomas is debated and not always supported by recent meta-analyses [8,9,15], in our protocol, the drain allowed for conservative management in one case of a minor hematoma, thereby avoiding a return to the operating room.

Finally, such favorable results might be partially attributable to surgeon experience and hospital volume, which are determinant variables for the achievement of favorable outcomes and patient safety [1]. In the present study, the surgeons performing the thyroid procedure were already considered experienced, potentially introducing a bias and possibly masking the positive effect produced by the standardized protocol. However, the standardization of the technique proposed here ensures that these results reproducible, even for less experienced surgeons, potentially helping to improve surgical outcomes.

Notwithstanding the encouraging outcomes, several limitations of this study must be acknowledged. Firstly, the observational single-arm design of this study is a limitation. Secondly, the sample size of 123 patients, while representative for a single center over an 18-month period, may not be sufficient to capture extremely rare “black swan” events. Additionally, historical results, including data prior to the application of the standardized surgical protocol, were not available, making a comparison impossible as well as demonstrating an improvement in surgical outcomes. Thirdly, the follow-up period was limited to one week after surgery, possibly excluding delayed post-operative bleeding. Fourthly, the single-center nature of this study raises questions regarding external validity.

Despite these constraints, we believe that the high degree of protocol adherence and the prospective data collection provide a solid foundation for future multicenter trials aimed at further refining safety standards for the prevention of hemorrhage following thyroid surgery.

## 5. Conclusions

In conclusion, the adoption of a standardized clinical pathway demonstrated favorable post-operative outcomes, especially for life-threatening events such as post-operative bleeding. The integration of hybrid energy device technology with traditional hemostasis maneuvers and strict hemodynamic control allowed us to achieve a zero re-operation rate for bleeding in our series. Such a standardized protocol may offer a foundation for building future surgical models to optimize patient safety within the challenging landscape of endocrine surgery. Studies including a control group or comparing surgical outcomes before and after applying the present protocol should be considered to confirm a significant benefit.

## Figures and Tables

**Table 1:** Characteristics of included patients.

Clinical Characteristics		
Patient characteristics	Age, y [mean (SD)]	57.3 (13.9)
	Female [n (%)]	95 (77.2)
	Comorbidity [n (%)]	81 (65.9)
Indication for surgery [n (%)]	Benign goiter	64 (52)
	TIR 3A or 3B	27 (22)
	TIR 4 or 5	13 (10.6)
	Toxic adenoma	4 (3.2)
	Graves’ disease	15 (12.2)
Symptomatic [n (%)]	All symptoms	67 (54.5)
	Compression symptoms	51 (41.5)
	Tracheal deviation	38 (30.9)
	Hyperthyroidism	35 (28.5)
Pre-operative TSH, mUI/L [n (%)]	<0.4	20 (16.5)
Pre-operative fT3, pmol/L [n (%)]	Above the limit	0
	Normal	17 (100)
Pre-operative fT4, ng/dL [n (%)]	Above the limit	2 (11.8)
	Normal	15 (88.2)
Type of surgery [n (%)]	Total thyroidectomy	83 (67.5)
	Hemithyroidectomy	40 (32.5)

**Table 2:** Surgical outcomes after thyroidectomy.

Surgical Outcomes	
Post-operative hypocalcemia, [n (%)]	11 (8.9)
Persistent hypoparathyroidism, [n (%)]	1 (1.35)
Monolateral post-operative cord palsy, [n (%)]	2 (1.6)
Re-operation for post-operative hemorrhage, [n (%)]	0
Post-operative hematoma, [n (%)]	1 (0.8)
Seroma, [n (%)]	6 (5)
Surgical site infection, [n (%)]	1 (1.1)

## Data Availability

The data presented in this study are available on request from the corresponding author.

## Supplementary Materials

The following supporting information can be downloaded at https://www.mdpi.com/article/10.3390/jcm15155801/s1: Table S1: Histological features of thyroid specimens after thyroidectomy.

## Abbreviations

The following abbreviations are used in this manuscript:

SSI	Surgical site infection
SD	Standard deviation
POD	Post-operative day

## References

[1] Sosa J.A., Bowman H.M., Tielsch J.M., Powe N.R., Gordon T.A., Udelsman R. (1998). The importance of surgeon experience for clinical and economic outcomes from thyroidectomy. Ann. Surg..

[2] Calò P.G., Pisano G., Piga G., Medas F., Tatti A., Donati M., Nicolosi A. (2010). Postoperative hematomas after thyroid surgery. Incidence and risk factors in our experience. Ann. Ital. Chir..

[3] Edafe O., Cochrane E., Balasubramanian S.P. (2020). Reoperation for Bleeding After Thyroid and Parathyroid Surgery: Incidence, Risk Factors, Prevention, and Management. World J. Surg..

[4] Fan C., Zhou X., Su G., Zhou Y., Su J., Luo M., Li H. (2019). Risk factors for neck hematoma requiring surgical re-intervention after thyroidectomy: A systematic review and meta-analysis. BMC Surg..

[5] Cannizzaro M.A., Bianco S.L., Picardo M.C., Provenzano D., Buffone A. (2017). How to avoid and to manage post-operative complications in thyroid surgery. Updates Surg..

[6] Cernea C.R., Brandão L.G., Hojaij F.C., De Carlucci D., Montenegro F.L., Plopper C., Vanderlei F., Gotoda R., Dias F.L., Lima R.A. (2010). How to minimize complications in thyroid surgery?. Auris Nasus Larynx.

[7] Revelli L., Damiani G., Bianchi C.B.N.A., Vanella S., Ricciardi W., Raffaelli M., Lombardi C.P. (2016). Complications in thyroid surgery. Harmonic Scalpel, Harmonic Focus versus Conventional Hemostasis: A meta-analysis. Int. J. Surg..

[8] Hurtado-López L.M., López-Romero S., Rizzo-Fuentes C., Zaldívar-Ramírez F.R., Cervantes-Sánchez C. (2001). Selective use of drains in thyroid surgery. Head Neck.

[9] Memeh K., Azar S.A., Afolaranmi O., Vaghaiwalla T.M. (2025). Practice variations, trends, and outcomes of drain use in thyroidectomy: A NSQIP study. Am. J. Surg..

[10] Iliff H.A., El-Boghdadly K., Ahmad I., Davis J., Harris A., Khan S., Lan-Pak-Kee V., O’cOnnor J., Powell L., Rees G. (2022). Management of haematoma after thyroid surgery: Systematic review and multidisciplinary consensus guidelines from the Difficult Airway Society, the British Association of Endocrine and Thyroid Surgeons and the British Association of Otorhinolaryngology, Head and Neck Surgery. Anaesthesia.

[11] Zhang X., Hu B., Xiao J., Zhang X., Zhang J., Zhu L., Kuang Y., Huang Z., Weng Y. (2025). Clinical features and nursing strategies of reexploration for hematomas after thyroid surgery: Insights from a 7-year single-center study in China. BMC Surg..

[12] Lee M., Rhee J., Kim Y., Jung Y.H., Ahn S.H., Jeong W.J. (2019). Perioperative risk factors for post-thyroidectomy hematoma: Significance of pain and ketorolac usage. Head Neck.

[13] Canu G.L., Medas F., Podda F., Tatti A., Pisano G., Erdas E., Calò P.G. (2020). Thyroidectomy with energy-based devices: Surgical outcomes and complications-comparison between Harmonic Focus, LigaSure Small Jaw and Thunderbeat Open Fine Jaw. Gland Surg..

[14] Montori G., Botteri E., Ortenzi M., Gerardi C., Allocati E., Giordano A., Vettoretto N., Arezzo A., Huo B., Bergamini C. (2024). Systematic review and meta-analysis of the use of high-energy devices for thyroid surgery. Langenbecks Arch. Surg..

[15] Khanna J., Mohil R.S., Chintamani, Bhatnagar D., Mittal M.K., Sahoo M., Mehrotra M. (2005). Is the routine drainage after surgery for thyroid necessary? A prospective randomized clinical study [ISRCTN63623153]. BMC Surg..

