# Supplementary material for: Thyroidectomy: A Standardized Surgical Technique with a Hybrid Energy Device to Minimize the Risk of Post-Operative Hemorrhage
Source: J Clin Med. 2026 Jul 24;15(15):5801. doi: 10.3390/jcm15155801 (PMC13466855; doi:10.3390/jcm15155801)
Supplement: Supplementary file 1 [file jcm-15-05801-s001.zip › jcm-4254904-supplementary.pdf]

Supplementary Table S1. Histological features of thyroid specimens after thyroidectomy.

**Histological characteristics**	
Malignant disease [n (%)]	18 (22.5)
Thyroiditis in the specimen [n (%)]	18 (23.1)
Thyroid weight, g [mean (SD)]	51.1 (46.3)
Tumor diameter, mm [mean (SD)]	54.7 (20.5)
